# *CMTR*—Aims and Scope Update

**DOI:** 10.3390/cmtr18040049

**Published:** 2025-11-27

**Authors:** Kathleen Fan, Yiu Yan Leung, Florian M. Thieringer

**Affiliations:** 1 King’s College Hospital NHS Foundation Trust, London SE5 9RS, UK; kathy.fan@kcl.ac.uk; 2 King’s College London, University of London, London WC2R 2LS, UK; 3 Discipline of Oral and Maxillofacial Surgery, Faculty of Dentistry, The University of Hong Kong, Hong Kong SAR, China; 4 Oral- and Cranio-Maxillofacial Surgery, University Hospital Basel, 4031 Basel, Switzerland; 5 Swiss MAM Research Group, Department of Biomedical Engineering, University of Basel, 4123 Basel, Switzerland

The journal *Craniomaxillofacial Trauma & Reconstruction* (*CMTR*) (ISSN 1943-3883) was launched in 2008 and officially transferred to MDPI in 2025. Since then, *CMTR* has published numerous valuable manuscripts covering the study and treatment of craniomaxillofacial conditions.

We are updating our Aims and Scope to ensure closer alignment with the research currently published in the journal and to better reflect the developments within our field.

This update will help us better serve our readers, authors, and the wider scientific community by providing clearer guidance and expanded space for publications in key and emerging areas of craniomaxillofacial research and clinical practice.

After a careful review of previously published articles and ongoing advancements in the disciplines relevant to *CMTR*, we have expanded the journal’s scope. More details are presented in Table 1.

## Figures and Tables

**Table 1:** Changes in the aims and scope of the journal *CMTR*.

Aims (New Version)	Aims (Old Version)
*Craniomaxillofacial Trauma & Reconstruction* (*CMTR*) (ISSN 1943-3883) is the official scientific publication of AO CMF. *CMTR* is a peer-reviewed, open access journal devoted to the study and the management of craniomaxillofacial conditions, including but not limited to the epidemiology, diagnosis, operative and non-operative management options, surgical techniques, emerging research, basic, translational, and clinical research in regenerative surgery and tissue regeneration, including bone, cartilage, and soft tissue repair, evidence-based therapy, and clinical developments. With a multi-disciplinary scope, *CMTR* features original research, reviews, commentaries, editorials, and technical reports (though other article types will be considered) pertinent to all specialties practicing craniomaxillofacial surgery. The journal promotes communication among oral and maxillofacial surgeons, otolaryngologists/head and neck surgeons, plastic surgeons, ophthalmologists/oculoplastic surgeons, and trauma surgeons across the globe by providing an integrated and balanced view of the current clinical science in craniomaxillofacial surgery. All results must be reproducible, and we strongly encourage the inclusion of data, code, and methodological transparency to support reproducibility. *CMTR* has no restrictions on the maximum length of manuscripts, provided that the text is concise and comprehensive.	*Craniomaxillofacial Trauma & Reconstruction (CMTR)* (ISSN 1943-3883) is the official scientific publication of AO CMF. A peer-reviewed journal, *CMTR* is devoted to the study and treatment of craniomaxillofacial conditions, including diagnosis, operative and non-operative treatment options, surgical techniques, emerging research, evidence-based therapy, and clinical developments. With a multi-disciplinary scope, *CMTR* features original research, reviews, commentaries, editorials, and technical reports pertinent to all specialties practicing craniomaxillofacial surgery. The journal promotes communication among oral and maxillofacial surgeon, otolaryngologists/head and neck surgeons, plastic surgeons, ophthalmologists/oculoplastic surgeons, and trauma surgeons across the globe by providing an integrated and balanced view of the current clinical science in craniomaxillofacial surgery.
**Scope (New Version)**	**Scope (Old Version)**
Craniomaxillofacial Pathology Craniomaxillofacial Trauma Dentoalveolar Surgery Dental Implant Rehabilitation Facial Aesthetic Surgery Hard and Soft Tissue Reconstruction of Craniomaxillofacial Defects Oculoplastic Surgery Orthognathic Surgery and Facial Deformities Pediatric Cleft and Craniofacial Surgery Management of Temporomandibular Disorders Management of Obstructive Sleep Apnea Head and Neck Surgery Regenerative Surgery and Tissue Regeneration	Craniomaxillofacial Pathology Craniomaxillofacial Trauma Dentoalveolar Surgery Dental Implant Reconstruction Facial Aesthetic Surgery Hard and Soft Tissue Reconstruction of Craniomaxillofacial Defects Oculoplastic Surgery Pediatric Cleft and Craniofacial Surgery

